# The Effects of Freezing Media on the Characteristics of Male and Female Chicken Primordial Germ Cell Lines

**DOI:** 10.3390/life13040867

**Published:** 2023-03-23

**Authors:** András Ecker, Bence Lázár, Roland Imre Tóth, Martin Urbán, Nikolett Tokodyné Szabadi, Maria Teresa Salinas Aponte, Mohd Adnan, Eszter Várkonyi, Elen Gócza

**Affiliations:** 1 Department of Animal Biotechnology, Institute of Genetics and Biotechnology, Hungarian University of Agriculture and Life Sciences, 2100 Gödöllő, Hungary; 2 Agribiotechnology and Precision Breeding for Food Security National Laboratory, 2100 Gödöllő, Hungary; 3 National Centre for Biodiversity and Gene Conservation, 2100 Gödöllő, Hungary

**Keywords:** chicken, cryopreservation, freezing medium, PGC, RNA expression

## Abstract

Recently, in vitro gene preservation has gained ground thanks to its lower cost and higher stability compared to in vivo techniques. One of the methods that can preserve female-specific W chromosome-linked genes is primordial germ cell (PGC) freezing. PGCs can be isolated from Hamburger–Hamilton stage 14–16 embryos via blood sampling. In our experiment, we used two newly established Black Transylvanian naked neck chicken cell lines and four cell lines from our gene bank. We compared two different freezing media (FAM1 and FAM2) in this study. The cell number and viability of the PGCs were measured before freezing (BF) and after thawing on Day 0, Day 1, and Day 7 of cultivation. We analyzed the germ cell-specific chicken vasa homologue (CVH) expression profile in PGCs using RT-qPCR. We found that on Day 0, immediately after thawing, the cell number in cell lines frozen with the FAM2 medium was significantly higher than in the FAM1-treated ones. On Day 1 and Day 7, the cell number and viability were also higher in most cell lines frozen with FAM2, but the difference was insignificant. The freezing also affected the chicken vasa homologue gene expression in male lines treated with both freezing media.

## 1. Introduction

The population of the world consumes more than 90 million tons of poultry meat per year, and this number is still growing [1]. To generate this amount of meat products, breeders need to evolve their stock’s production rate. Because of this, many breeders turn to modern breeds and hybrids with huge production rates and easy-to-mechanize, intensive keeping. As a side effect, the traditional or local breeds often merge into these new hybrids or become extinct because of their loss of space due to the modern types.

However, these traditional breeds carry important and valuable alleles and attributes. For instance, Transylvanian naked neck breeds have outstanding resistance against heat stress. Such abilities might play a crucial role in the future to adapt the chicken population to the changing climate or new diseases. These local types could also bring some genetic diversity to the inbred industrial stocks. Alongside these reasons, the cultural legacy of these animals must also be considered.

The genetic information of avian species must be preserved, although there are challenges that need to be overcome to reach this goal. Nowadays, sperm freezing is widely used among poultry species, although its effectiveness still needs improvement. Furthermore, this technique is more suitable for mammalian species due to the homogametic nature of male birds. In mammals, males have a heterogametic chromosome pair (XY), and females are of the homogametic sex (XX), whereas in avians, the opposite is true: males have ZZ, and females have ZW sex chromosome pairs. According to this, with sperm freezing, only Z-chromosome-linked genes can be preserved, and W-chromosome-linked genes are lost. Thus, another method needs to be found and applied parallel to sperm freezing for avian gene preservation.

One of the solutions to this issue could be ovary and testis tissue transfers in day-old chicks [2,3,4,5]. It is relatively easy to isolate these tissues from donor animals, and they have a great integration ability with the recipient organism. However, this method cannot be used in endangered or valuable species, because the donor animals do not survive this procedure.

A great alternative to this problem might be primordial germ cell-based techniques. Primordial germ cells (PGCs) are the precursor cells of germ cells, which means they have a unipotent ability to develop into sperm or egg cells. They can differentiate both in vivo and in vitro [6]. They are present in the embryo by the time the primitive streak forms [7]. At this stage, the cells are in the ventral side of the epiblast layer, but after 18 h of incubation, they migrate to the germinal crescent [8,9]. Here, they start to proliferate, and by the 40th hour, they enter the blood circulation and migrate to the place of the gonads (Hamburger–Hamilton stage 12–17), where they are then colonized [7,8,10]. The cPGCs are easier to isolate from the embryo’s bloodstream and do not necessarily require the death of the donor embryo. According to previous results, they can be preserved in a 10% cHanks’ solution–dimethyl sulfoxide (DMSO) mixture with a 94,2% viability among the cells after freezing [11]. This research aims at finding an even more simplified medium with possibly higher viability rates.

Primordial germ cells can be used in various fields of science: subjects for cryopreservation [11,12,13,14,15], tools for transgenesis and chimera production [16,17,18,19], and models for reproductive system development [20]. This is due to their relatively easy isolation protocol, feasible culturing, and greatly inductive nature into the donor organism. In this experiment, the PGCs’ gene conservational potential was harnessed.

Previously, many researchers mentioned PGC freezing as a promising technique for gene preservation [21,22]. There have been several occasions with several types of media used for the cryopreservation of these cells. The usual freezing medium contains serum and 10% DMSO, although media with more than 10% serum and 5–10% DMSO or 10% ethylene glycol as cryoprotectants had higher recovery and viability rates [23]. However, including chicken serum into the medium ruins the probability of universal usage for multiple species. DMSO was proven to be the optimal cryoprotectant base for chicken primordial germ cells [13].

Gonadal PGCs can be preserved by creating a cell suspension via trypsin digestion and frozen in MEM containing 10% DMSO [18]. PGC-containing stage X blastoderms can also be involved in gene preservation. After the trypsin treatment of the area pellucida, the blastodermal cells (with the PGCs among them) can be frozen in DMEM containing 30% PBS and 20% DMSO [12,16]. Unfortunately, both of these methods have relatively low effectiveness, because PGCs are mixed with non-germline compatible cells. Furthermore, these methods need many more steps than working with circulating PGCs (cPGCs) and certainly require the death of the donor animal.

The two freezing media used in this study originated from Whyte [24] and Kong [25]. These media were chosen due to their effectiveness and reliability in previous experiments. The former (FAM1) uses more ingredients, which makes it more specific, whereas the second (FAM2) is more universal and has a more basic composition. The main goal of this examination was to compare these two media according to the cell number, viability rate, and RNA expression and to make recommendations for further research applications based on the results.

## 2. Materials and Methods

### 2.1. Ethics Statement

Animals were kept and maintained according to general animal welfare prescriptions of the Hungarian Animal Protection Law (1998; XXVIII). All experimental methods described herein were approved by the Institutional Ethics Review Board of the Institute for Farm Animal Gene Conservation (no. 7/2011).

### 2.2. Maintenance of Domestic Fowl Experimental Stocks

The breed used in this experiment was the Black Transylvanian naked neck chicken. The animals were kept at the National Centre for Biodiversity and Gene Conservation in Gödöllő, Hungary. The old Hungarian chicken breeds were kept in barns with large outdoor areas in the institute. The stocking density is 5–6 birds/m^2^, and the sex ratio is 7 hens with 1 cockerel. There were nest boxes (5 hens/nest) for the collection of eggs. Breeding flocks were fed with laying mash in addition to limestone grit. The eggs were collected twice a day and then stored in a refrigerated room. The eggs were transported to the Institute of Genetics and Biotechnology of the Hungarian University of Agriculture and Life Sciences, where the experiment took place.

### 2.3. Isolation, Establishment, and Maintenance of PGC Lines

Incubation was carried out with a MIDI F500S hatchery machine (PL Machine Ltd., Tárnok, Hungary) with two 45° rotations per hour. The incubation temperature was 37.8 °C with 70% relative humidity. When they reached the 51–56 h age (Hamburger–Hamilton stage 16 embryos), they were opened, and the embryos’ blood was harvested via blood sampling. The blood samples were taken through the dorsal aorta using a mouth pipette and a glass microcapillary. Approximately 1 µL blood was collected from each embryo individually.

The blood was blown into the culturing medium on a 48-well plate. The culture medium consisted of calcium-free DMEM (Gibco, Billings, MT, USA, 21068-028), tissue culture-grade water (Gibco, Billings, MT, USA, A12873-01), pyruvate (Gibco, Billings, MT, USA, 11360039), MEM vitamin solution (Gibco, Billings, MT, USA, 11120052), MEM amino acids (Sigma, St. Louis, MO, USA, M5550), a B27 supplement (Gibco, Billings, MT, USA, 17504044), glutamax (Gibco, Billings, MT, USA, 35050038), nonessential amino acids (Gibco, Billings, MT, USA, 11140035), nucleosides (EmbryoMax, Munich, Germany, ES-008-D), B-mercaptoetanol (Gibco, Billings, MT, USA, 31350010), CaCl_2_ (Sigma, St. Louis, MO, USA, C4901-100G), ovalbumin (Sigma, St. Louis, MO, USA, A5503), Na heparin (Sigma, St. Louis, MO, USA, H3149-25KU), a penicillin–streptomycin mixture (Gibco, Billings, MT, USA, 15070-063), chicken serum (Sigma, St. Louis, MO, USA, C5405), human activin (Invitrogen, Waltham, MA, USA, PHC9564), bFGF2 (Gibco, Billings, MT, USA, 13256-029), and ovotransferrin (Sigma, St. Louis, MO, USA, C7786). [24] The cell lines were cultured with a Monday-Wednesday-Friday medium-changing schedule. The embryos were collected for DNA isolation and sex determination. According to this, two male (M1, M2) and two female (F1, F2) cell lines were chosen for the viability and cell number tests, and a male (A1) and a female (A2) were chosen for the immunostaining and the microRNA examinations. To check the potential of the PGC gene bank samples, the M and F lines were chosen from the gene bank in Gödöllő; however, this excluded them from the RNA comparison measurements, since freshly isolated lines were recommended for those. Due to this, the A1 and A2 lines were isolated for this part of the study. The sex was identified by PCR using the embryonic tissue.

### 2.4. Freezing and Thawing of PGC Lines

After four weeks of culturing, the cell lines reached the optimal cell number (80,000 cell/300 uL medium) and purity, which was detected by the NanoEntek Arthur Novel Fluorescence Cell Counter (NanoEntek, Seoul, Korea) according to the cells’ shape and size. Two media were used for freezing: FAM1 (containing DMEM (Gibco, Billings, MT, USA, 21068-028) and water (Gibco, Billings, MT, USA, A12873-01) mixed in a 2:1 ratio, sodium pyruvate (Gibco, Billings, MT, USA, 11360039), 10% chicken serum (Sigma, St. Louis, MO, USA, C5405), CaCl_2_ (Sigma, St. Louis, MO, USA, C4901), and 16% DMSO (Sigma, St. Louis, MO, USA, D2650)) [24] and FAM2 (containing only FBS (Gibco, Billings, MT, USA, 10108-165) and 20% DMSO (Sigma, St. Louis, MO, USA, D2650)) [25]. For the detailed recipes of the freezing media, see Table 1.

For freezing, the cells were collected and centrifuged. They were resuspended in 250 µL 2:1 DMEM–water mixture. Then, 250 µL freezing medium was slowly added to them to produce the final DMSO concentrations (8% for FAM1 and 10% for FAM2). The samples were quickly put into a Mr. Frosty^TM^ Freezing Container (Thermo Fischer, Waltham, MA, USA) to ensure controlled freezing and then transferred into a −80 °C freezer, so the DMSO could not harm the cells. Sixteen samples were frozen, two from each line in both freezing mediums.

On Day 0 of the experiment, the samples were thawed at room temperature (RT), and they were immediately added into 900 µL culture medium. The samples were centrifuged to get rid of the DMSO and resuspended in 300 µL culture medium before adding them to the culturing plates. The medium was changed on Day 1, Day 3, and Day 6. The experiment was repeated by thawing the parallel samples.

### 2.5. Measurements of Cell Number and Viability of PGC Lines

A NanoEntek Arthur Novel Fluorescence Cell Counter (NanoEntek, Soeul, Republic of Korea) was used for the cell number and viability measurements. The measurements were performed before freezing and on Day 0, Day 1, and Day 7 after thawing. An amount of 20 µL cell suspensions were transferred into 1.5 mL tubes and stained with 1 µL propidium iodide (PI Solution) from the Annexin V, FITC Apoptosis Detection Kit (Dojindo, Munich, Germany, AD10). The samples were incubated at room temperature and protected from light for 15 min. After that, 80 µL of 1xPBS was added to the samples to stop the staining.

Two amounts of 25 µL of each sample were added to the Arthur slides, and then the slides were measured (each slide has two sides for parallel measurements). Before each addition, the cells were suspended thoroughly to reach the optimal homogenous suspension. Cells between 7 and 25 µm in diameter were counted [26]. This size was based on previous measurements using confocal imaging with a Leica TCS SP8 Confocal Microscope (Leica, Wetzlar, Germany).

### 2.6. DNA Isolation and Sex Determination

For isolating the DNA, the High Pure PCR Template Preparation Kit (Roche Diagnostics, Indianapolis, IN, USA) was used according to the manufacturer’s instructions. The sex of the donor embryos and the established PGC lines were determined with the P2–P8 primer set, as described before by Griffiths and colleagues [27]. The isolated DNA was diluted to a 25 ng/μL concentration for PCR reaction and gel electrophoresis. MyTaq Red Mix was used for the reaction (Bioline Reagents Ltd., London, UK). The PCR products were then separated by electrophoresis, using 3% agarose gel stained with ethidium bromide at 100 V for 1.5–2.0 h. The DNA bands were then visualized under UV illumination and photographed [28].

### 2.7. RNA Isolation and Synthesis of cDNA

For RNA isolation, the A1 (ZZ) and A2 (ZW) lines were used. Samples were collected from the freshly established cultures and from the cultures that had been frozen with FAM1 or FAM2 medium. At the beginning of RNA isolation, 125 µL of pure ethanol was added to the samples. After this step, the RNAs were isolated according to the protocol of the RNAquarious^TM^-Micro Total RNA Isolation Kit (Thermo Fischer Scientific, Waltham, MA, USA, AM1931). The isolated RNA concentration was measured with a NanoDrop One Spectrophotometer (Thermo Scientific, Waltham, MA, USA). The samples were then diluted to a 25 ng/ µL concentration.

An amount of 15 µL from the RNA samples was put into the tubes, and 15 µL Master Mix was added to them. The Master Mix was prepared according to the High-Capacity cDNA Reverse Transcription Kit (Applied Biosystem, Waltham, MA, USA, 4368814). The PCR lasted 135 min: 10 min preheating at 25 °C, 120 min incubation at 37 °C, and a 5-min heat treatment at 85 °C.

For microRNA measurements, the RNA samples were diluted to 5 ng/ µL, and the cDNA was written with the TaqMan^®^ MicroRNA Reverse Transcription Kit (Applied Biosystems, Waltham, MA, USA, PN: 4366596). The examined micro RNAs were hsa-miR-92 (000430, Lot: P210512-001 E10), hsa-miR-302b (000531, Lot: P211022-000 H05), and gga-miR-302b* (008131, Lot: P201108-001 A12). The same machine was used for microRNA expression measurements. The run consisted of a 30-min-long 16 °C first phase, a 30-min-long 42 °C and a 5-min-long 85 °C second phase, and a 4 °C third phase, which had no time limit.

### 2.8. qPCR

After the cDNAs were produced, the samples were prepared for qPCR. The mix for one sample contained 5.75 µL nuclease-free water, 7.5 µL Power SYBRGreen PCR Master Mix (Applied Biosystems, 4368575), 0.75 µL from both forward and reverse primer, and 0.5 µL cDNA suspension. Three types of primer were used: glyceraldehyde-3-phosphate dehydrogenase (GAPDH; Integrated DNA Technologies, Coralville, IA, USA) for control and chicken vasa homolog (CVH; Integrated DNA Technologies, Coralville, IA, USA) and Deleted in Azoospermia Like gene primer (DAZL; Integrated DNA Technologies, Coralville, IA, USA) for germ cell specificity. The qPCR program was performed in a Mastercycler^®^ Realplex Real-Time PCR System (Eppendorf, Hamburg, Germany) and consisted of a 10 min 95 °C first phase, a 15 s 95 °C, 40 s 60 °C, and 20 s 68 °C second phase with 40 repetitions, and a third phase containing a 15 s 95 °C, 15 s 48 °C, and 15 s 95 °C part with a 10 min heating stage before the last state. ROX was used as the reference dye.

In the case of qPCRs performed with cDNA synthesized for miRNAs, for 1 µL of each cDNA sample, a Master Mix was added consisting of 5.75 µL nuclease-free water (Ambion, Austin, TX, USA, AM9938), 7.5 µL TaqMan^®^ Universal Master Mix II with UNC (Applied Biosystems, Waltham, MA, USA, 4427983), and the TM primer pair for the currently used microRNA marker. The run consisted of a 10-min-long 95 °C phase and a 20-s-long 95 °C plus 65-s-long 60 °C phase with 40 repetitions.

### 2.9. Immunohistochemical Staining of PGCs

For immunohistochemical staining, 10 µL PGC containing PBS (Gibco, Billings, MT, USA, 14190-144) drops with 0.1% BSA (Sigma, St. Louis, MO, USA, A3311) were placed on glass microscope slides. The samples were fixed to the slide with a 4% PFA solution (Fluka, Buchs, Switzerland, 30525-89-4). Blocking solution (PBS with 0.1% BSA, 0.1% Triton-X-100 (Fluka, Buchs, Switzerland, 93426), and 2,5% donkey serum) was added to the drops to block the unnecessary expression sites and make the cell membrane permeable for the antibodies to stain the cytoplasm and the nucleus.

Stem cell-specific anti-SSEA-1 (Millipore, Munich, Germany, MC480) and primordial germ cell-specific anti-CVH (chicken vasa homolog, kindly provided by Bertrand Pain from the Stem cell and Brain Research Institute (SBRI), Lyon, France) primary antibodies were used for the staining. Red-colored Anti-Mouse-IgM-rD549^®^ (Jackson ImmunoResearch, West Grove, PA, USA, 715-505-140) was attached to the SSEA-1, and green Alexa Fluor^®^ 488 Anti-Rabbit-IgG (H+L) (Life Technologies/Molecular Probes, Carlsbad, CA, USA, A-21207) was added to stain the CVH. TO-PRO™-3 iodide (642-661)(Invitrogen, Waltham, MA, USA, T3605) far-red nucleus stain was used to mark the nuclei of the cells. The samples were covered with Vectashield Mounting Medium for Fluorescence (Vector, Burlingame, CA, USA, H-1000) and a cover glass. The edges of the cover glass were glued to the slide. The inspection of the samples was performed with a confocal microscope.

### 2.10. Statistical Analysis

The cell number and viability results were analyzed with Microsoft Excel and RStudio (1.0.136) software. The averages from the parallel measurements were compared between the two media in the cases of both cell number and viability. The results were also compared on different days. Two-sampled *t*-tests were used to analyze the differences between the groups. Levels of significance were applied as follows: * *p* < 0.05, ** *p* < 0.01, and *** *p* < 0.001.

For the qPCR and microRNA results, the analysis was started in Eppendorf Realplex software (Eppendorf, Hamburg, Germany) to sort the data. After that, GenEX software (MultiD Analyses AB, Göteborg, Sweden) was used to compute the averages, compare the samples to the GAPDH control and the control samples, and to test the significance between the pre-frozen and post-frozen samples, the two freezing media groups, and the two sexes.

## 3. Results

### 3.1. Cell Number Measurements

**Figure 1:**
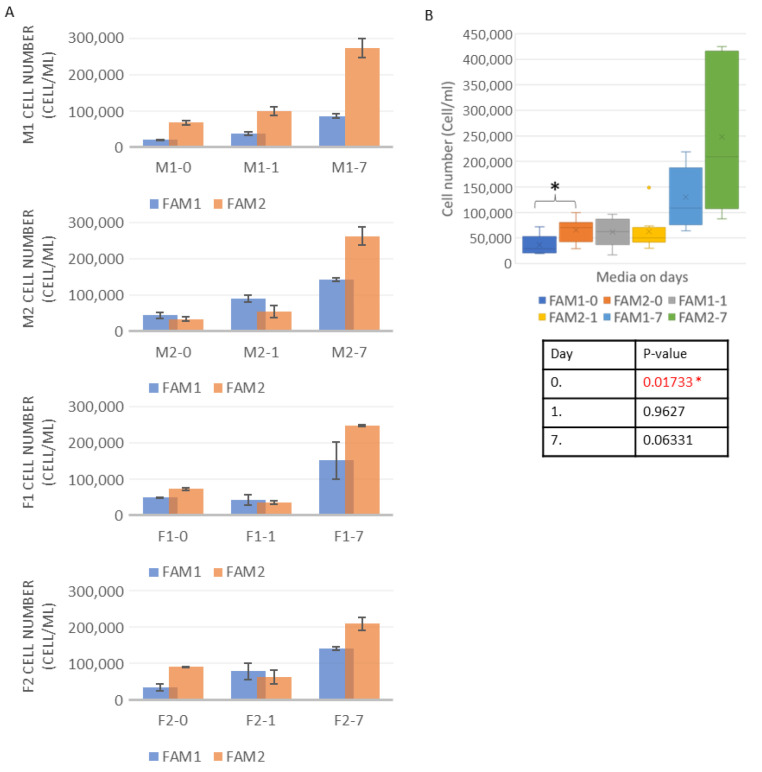
Cell numbers with different freezing media were compared (**A**). The results showed that in most cases, the FAM2 medium produced better cell numbers. All the data were averaged on the different days with the different media, and the results were examined with two-sampled T-probe in RStudio software (**B**). This revealed that on Day 0, there was a significant difference between the two freezing media in the cell number produced. The significant data is marked with a star in the Figure.

The cell number was measured before adding the freezing media to the cultures. This was necessary to set a baseline cell number to which the data received after the thawing could be compared. The cell counting showed that the F1 (4.16 × 10^5^ cell/mL) and the M1 (4.04 × 10^5^ cell/mL) lines had the highest concentration, and the F2 line had the lowest (2.27 × 10^5^ cell/mL). Between the two media, the statistical analysis showed a *p* = 0.01733 value on Day 0, a *p* = 0.9627 value on Day 1, and a *p* = 0.06331 value on Day 7. The complete cell number dataset is shown in Figure 1.

### 3.2. Cell Viability Measurements

Healthy cell lines were needed to make sure that enough cells remained alive even after the freezing loss. The viability test revealed that the female lines had higher viability (F1 and F2 both 97.5%), but the male lines’ quality was also acceptable (M1—96%; M2—93%).

**Figure 2:**
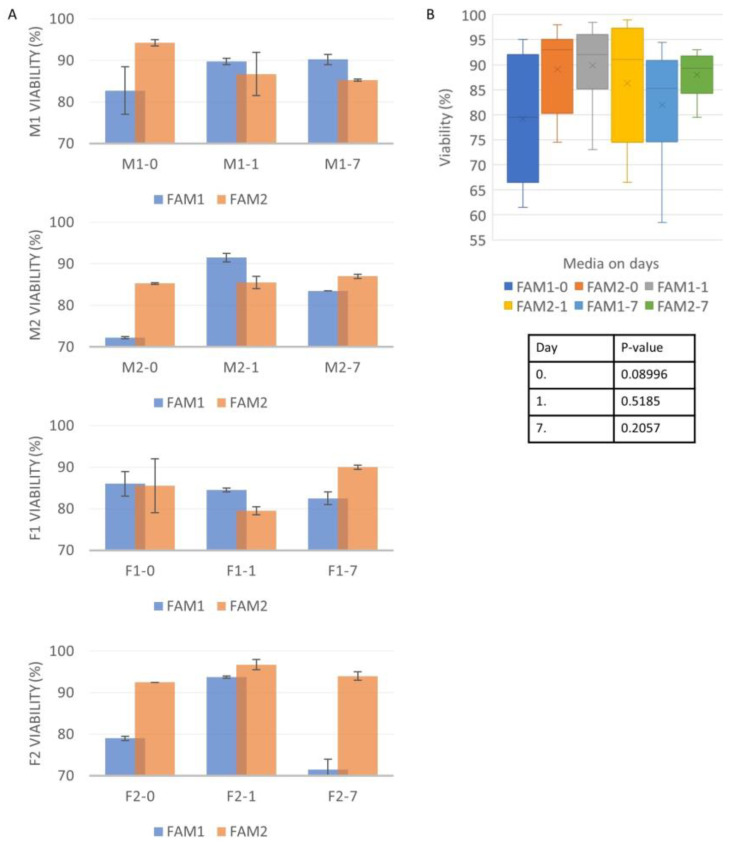
The cell viability with the different freezing media were compared (**A**). The results showed a more equal ratio between the two media; however, the FAM2 cultures produced better results. All the data were averaged on the different days with the different media, and the results were examined with two-sampled T-probe in RStudio software (**B**). After the statistical analysis, no significant difference was found between the two media.

After thawing on Day 0, the FAM2-treated lines had higher viability, except in the case of F1. On Day 7, the M2, F1, and F2 lines also favored the FAM2 medium. On Day 1, all the lines showed a FAM2 preference. The statistical analysis showed a *p* = 0.08996 value on Day 0, a *p* = 0.5185 value on Day 1, and a *p* = 0.2057 value on Day 7. There was an elevated standard deviation regarding most of the samples, which could have happened due to the low number of parallel measurements (Figure 2).

### 3.3. qPCR, MicroRNA Expression, and Immunohistochemical Staining

**Figure 3:**
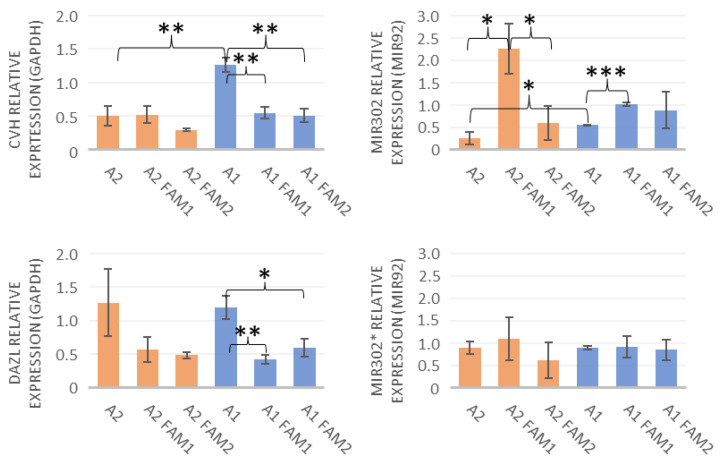
The expression of CVH and DAZL was compared to the GAPDH control level. The data labeled with the cell line names (A1, A2) show the BF results, and the FAM1 and FAM2 data present the data from after freezing. The results were analyzed via the GenEX software and showed multiple significant data. The most interesting observation was the reduction in CVH expression in the male line after freezing. It seems that in male PGCs, the cryopreservation lowered the CVH expression to a level equal to the female BF expression rate. * *p* < 0.05, ** *p* < 0.01, and *** *p* < 0.001.

Both the A1 (ZZ) and A2 (ZW) lines expressed the CVH and the DAZL. The male lines expressed higher levels of CVH due to its location on the Z chromosome. The miR-302 expression showed an opposite tendency compared to the CVH (Figure 3). The miR-302* data showed no clear tendency.

The statistical analysis compared the sexes before freezing (*p*-values are 0.006 for CVH, 0.865 for DAZL, 0.049 for miR-302, and 0.983 for miR-302*), as well as the before-freezing and after-thawing data (*p*-values are for A1 BF- A1 FAM1: 0.003 CVH, 0.006 DAZL, 0.0002 miR-302, 0.94 miR-302*; for A1 BF- A1 FAM2: 0.003 CVH, 0.024 DAZL, 0.357 miR-302, 0.805 miR-302*; for A2 BF- A2 FAM1: 0.926 CVH, 0.157 DAZL, 0.011 miR-302, 0.655 miR-302*; for A2 BF- A2 FAM2: 0.137 CVH, 0.107 DAZL, 0.339 miR-302, 0.426 miR-302*) and the two freezing media (*p*-values are for A1 FAM1- A1 FAM2: 0.731 CVH, 0.214 DAZL, 0.711 miR-302, 0.824 miR-302*; for A2 FAM1- A2 FAM2: 0.079 CVH, 0.588 CVH, 0.033 miR-302, 0.391 miR-302*).

**Figure 4:**
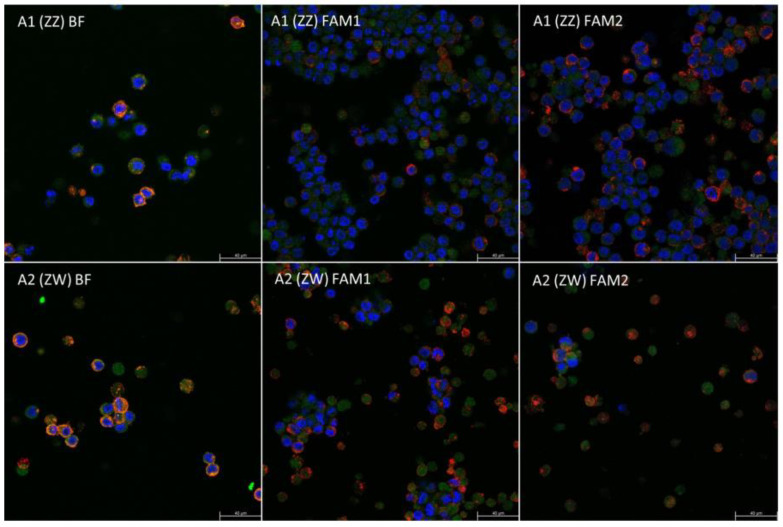
The immunohistochemical staining included a stem cell-specific SSEA-1 and a germ cell-specific CVH antibody. All cells were marked with the TO-PRO-3 nucleus stain. The SSEA-1 is expressed from the cell membrane with red, the CVH from the cytoplasm with green color. The TO-PRO-3 red nucleus stain is marked blue on the confocal images.

The immunohistochemical staining showed the presence of SSEA-1 in the cell membrane and CVH protein in the cytoplasm, proving that the cells were PGCs (Figure 4).

## 4. Discussion

PGCs are a promising tool for embryonic developmental models, animal healthcare, gene editing, and gene preservation in avian species [29]. In chickens, they are relatively easy to isolate from Hamburger–Hamilton 14–17 stage embryos [30], and a reliable culture medium has already been developed for them [24]. The circulating PGCs have an advantage compared to other cell types (gonadal tissues or gPGCs), in that their isolation method can be accomplished while sparing the life of the donor animal. To ensure the effective use of PGCs in in vitro methods, healthy cultures with high cell numbers are needed. This can be achieved by developing culture media. However, for gene preservation and other long-lasting techniques, the cells need to be conserved via freezing. There are already multiple freezing media that are proven to have little effect on the cells’ viability and proliferation after thawing. This experiment aimed to compare two of these media by their effects on cell number, viability, RNA, and microRNA expression before and after freezing.

The received data showed that the cells frozen with FAM2 freezing medium had a significantly higher cell number on Day 0. In most cases, FAM2 also had more promising results for cell numbers and viability, but no other significant differences could be found. This might have occurred due to the relatively high deviance in the parallel groups. Compared to other studies in which the 3% and 5% DMSO-DMEM medium produced only 82.4% and 68.3% average viability rates with month-long freezing and 81.6% and 49.7% average rates with 6-month-long freezing [31], this experiment achieved higher viability rates with both FAM1 and FAM2.

Since the isolation of the chicken vasa homologue (CVH) gene [32], this tool has been an effective marker of germ cell specificity. Its presence was proven during the early embryonic stages in PGCs [33] and was used to identify primordial germ cells [34,35] due to its high specificity, although recent studies showed that even the germ cell specificity of CVH is not total [36]. However, the effects of freezing on the expression of CVH have not been examined.

In the case of the A1 (ZZ) line, the expression of CVH was significantly reduced with both freezing media, which indicates that the freezing treatment lowers the expression of CVH in male lines to the same level that was measured in the female lines. The A2 (ZW) line expressed CVH on a constant level before and after freezing. The expression of DAZL was also reduced by freezing in both media, although this reduction was significant only in the case of A1 samples.

In the BF A1 (ZZ) line the miR302 expression showed a higher rate than in its female counterpart. In both sexes, the FAM1 lines were expressed on a higher level than the unfrozen cultures. In the case of the A2 (ZW) line, the FAM1 miR302 was expressed on a significantly higher level than the FAM2 line. The expression of miR-302* did not seem to be affected by freezing. These results were also mirrored by the female A2 line; however, no significant differences could be found in any of those data. Once again, this could be due to the occasionally high standard deviations.

The results between the cell groups frozen with different freezing media showed no significant differences. The comparison of the two sexes produced a significant difference only with the CVH, which can be explained by the chromosomal placement of the chicken vasa homolog gene [37]. The lack of significance could be due to the occasionally high deviation in some sample groups.

## Figures and Tables

**Table 1:** Components of the two freezing media.

**FAM1**	
Avian KO DMEM	3665 µL
Na Pyruvate	20 µL
Chicken serum 10%	500 µL
CaCl_2_	15 µL
DMSO	800 µL
**FAM2**	
FBS	4000 µL
DMSO	1000 µL

## Data Availability

The data presented in this study are available on request from the corresponding author.

## References

[1] Sumner J.H. Incredible goals of the world’s poultry industry. Proceedings of the International Poultry Day Conference.

[2] Barna J., Liptói K., Barna E., Liptói K., Patakiné Várkonyi E. (2016). Save what can be saved-new possibilities in in vitro gene preservation of poultry species (in Hungarian). Hungarian Vet. J..

[3] Liu J., Cheng K.M., Silversides F.G. (2013). Production of live offspring from testicular tissue cryopreserved by vitrification procedures in Japanese quail (*Coturnix japonica*). Biol. Reprod..

[4] Liu J., Cheng K.M., Silversides F.G. (2013). Fundamental principles of cryobiology and application to ex situ conservation of avian species. Avian Biol. Res..

[5] Silversides F.G., Robertson M.C., Liu J. (2013). Cryoconservation of avian gonads in Canada. Poult. Sci..

[6] Xiong H., Pu Y., Hu Q., Shan Z., Hu P., Guan W., Ma Y. (2015). Embryoid bodies formation from chicken primordial germ cells. Anim. Cells Syst..

[7] Eyal-Giladi H., Ginsburg M., Farbarov A. (1981). Avian primordial germ cells are of epiblastic origin. J. Embryol. Exp. Morphol..

[8] Kochav S., Ginsburg M., Eyal-Giladi H. (1980). From Cleavage to Primitive Streak Formation: A Complementary Normal Table and a New Look at the First Stages of the Development of the Chick II. Microscopic Anatomy and Cell Population Dynamics. Dev. Biol..

[9] Nakamura Y., Yamamoto Y., Usui F., Mushika T., Ono T., Setioko A.R., Takeda K., Nirasawa K., Kagami H., Tagami T. (2007). Migration and proliferation of primordial germ cells in the early chicken embryo. Poult. Sci..

[10] Ginsburg M., Eyal-Giladi H. (1986). Temporal and spatial aspects of the gradual migration of primordial germ cells from the epiblast into the germinal crescent in the avian embryo. J. Embryol. Exp. Morphol..

[11] Naito M., Tajima A., Tagami T., Yasuda Y., Kuwana T. (1994). Preservation of chick primordial germ cells in liquid nitrogen and subsequent production of viable offspring. J. Reprod. Fertil..

[12] Naito M. (2003). Cryopreservation of avian germline cells and subsequent production of viable offsprings. J. Poult. Sci..

[13] Setioko A.R., Tagami T., Tase H., Nakamura Y., Takeda K., Nirasawa K. (2007). Cryopreservation of premordial germ cells (PGCs) from White Leghorn embryos using commercial cryoprotectants. J. Poult. Sci..

[14] Nakamura Y., Usui F., Miyahara D., Mori T., Watanabe H., Ono T., Takeda K., Nirasawa K., Kagami H., Tagami T. (2011). Viability and functionality of primordial germ cells after freeze-thaw in chickens. J. Poult. Sci..

[15] Nandi S., Whyte J., Taylor L., Sherman A., Nair V., Kaiser P., Mcgrew M.J. (2016). Cryopreservation of specialized chicken lines using cultured primordial germ cells. Poult. Sci..

[16] Kino K., Pain B., Leibo S.P., Cochran M., Clark M.E., Etches R.J. (1997). Production of Chicken Chimeras from Injection of Frozen-Thawed Blastodermal Cells. Poult. Sci..

[17] Ono T., Matsumoto T., Arisawa Y. (1998). Production of Donor-Derived Offspring by Transfer of Primordial Germ Cells in Japanese Quail. Exp. Anim..

[18] Tajima A., Naito M., Yasuda Y., Kuwana T. (1998). Production of germ-line chimeras by transfer of cryopreserved gonadal primordial germ cells (gPGCs) in chicken. J. Exp. Zool..

[19] Trefil P., Aumann D., Koslová A., Mucksová J., Benešová B., Kalina J., Wurmser C., Fries R., Elleder D., Schusser B. (2017). Male fertility restored by transplanting primordial germ cells into testes: A new way towards efficient transgenesis in chicken. Sci. Rep..

[20] Bednarczyk M., Dunislawska A., Stadnicka K., Grochowska E. (2021). Chicken embryo as a model in epigenetic research. Poult. Sci..

[21] Petitte J.N. (2006). Avian germplasm preservation: Embryonic stem cells or primordial germ cells?. Poult. Sci..

[22] Liu J., Cheng K.M., Silversides F.G. (2012). Novel needle-in-straw vitrification can effectively preserve the follicle morphology, viability, and vascularization of ovarian tissue in Japanese quail (*Coturnix japonica*). Anim. Reprod. Sci..

[23] Nakamura Y. (2016). Poultry genetic resource conservation using primordial germ cells. J. Reprod. Dev..

[24] Whyte J., Glover J.D., Woodcock M., Brzeszczynska J., Taylor L., Sherman A., Kaiser P., McGrew M.J. (2015). FGF, Insulin, and SMAD Signaling Cooperate for Avian Primordial Germ Cell Self-Renewal. Stem Cell Rep..

[25] Kong L., Qiu L., Guo Q., Chen Y., Zhang X., Chen B., Zhang Y., Chang G. (2018). Long-term in vitro culture and preliminary establishment of chicken primordial germ cell lines. PLoS ONE.

[26] Zhao D.F., Kuwana T. (2003). Purification of avian circulating primordial germ cells by Nycodenz density gradient centrifugation. Br. Poult. Sci..

[27] Griffiths R., Double M.C., Orr K., Dawson R.J.G. (1998). A DNA test to sex most birds. Mol. Ecol..

[28] Lázár B., Anand M., Tóth R., Várkonyi E.P., Liptói K., Gócza E. (2018). Comparison of the MicroRNA expression profiles of male and female avian primordial germ cell lines. Stem Cells Int..

[29] Chaipipat S., Sritabtim K., Piyasanti Y., Prukudom S., Jurutha J., Phetpila V., Sinsiri R., Kammongkun J., Molee A., Thiangtum K. (2022). Initiative on Avian Primordial Germ Cell Cryobanking in Thailand. Biopreserv. Biobank..

[30] Hamburger V., Hamilton H.L. (1951). A series of normal stages in the development of the chick embryo. J. Morphol..

[31] Divya D., Shukla R., Chatterjee R., Sagar G., Rajendra Prasad A., Bhattacharya T. (2021). Production of Transgenic Chimeric Chicken from Cryopreserved Primordial Germ Cells and its Validation by Developing shRNA Transgenic Chicken Chimera. Res. Sq..

[32] Tsunekawa N., Naito M., Sakai Y., Nishida T., Noce T. (2000). Isolationof chicken vasa homolog gene and tracing the origin of primordial germ cells. Development.

[33] Kim Y.M., Han J.Y. (2018). The early development of germ cells in chicken. Int. J. Dev. Biol..

[34] Lázár B., Molnár M., Sztán N., Végi B., Drobnyák Á., Tóth R., Tokodyné Szabadi N., McGrew M.J., Gócza E., Patakiné Várkonyi E. (2021). Successful cryopreservation and regeneration of a partridge colored Hungarian native chicken breed using primordial germ cells. Poult. Sci..

[35] Ezaki R., Hirose F., Furusawa S., Horiuchi H. (2020). An improved protocol for stable and efficient culturing of chicken primordial germ cells using small-molecule inhibitors. Cytotechnology.

[36] Lejong M., Choa-Duterre M., Vanmuylder N., Louryan S. (2020). Is Vasa such a highly specific marker for primordial germ cells? A comparison of VASA and HSP90 proteins expression in young chicken embryos. Morphologie.

[37] Mazzoleni S., Němec P., Albrecht T., Lymberakis P., Kratochvíl P., Rovatsos M. (2021). Long-term stability of sex chromosome gene content allows accurate qPCR-based molecular sexing across birds. Mol. Ecol..

